# Microstructured Hyaluronic Acid Hydrogel for Tooth Germ Bioengineering

**DOI:** 10.3390/gels7030123

**Published:** 2021-08-18

**Authors:** Sol Park, Naomi W. Y. Huang, Cheryl X. Y. Wong, Jing Pan, Lamyaa Albakr, Jing Gu, Lifeng Kang

**Affiliations:** 1 School of Pharmacy, Faculty of Medicine and Health, University of Sydney, Pharmacy and Bank Building A15, Science Road, Sydney, NSW 2006, Australia; spar2750@uni.sydney.edu.au; 2 Department of Pharmacy, National University of Singapore, 18 Science Drive 4, Singapore 117543, Singapore; naomihuang@u.duke.nus.edu (N.W.Y.H.); e0020785@u.nus.edu (C.X.Y.W.); 3 Skinetrate Pte. Ltd., 79 Ayer Rajah Crescent, Singapore 139955, Singapore; pan.j@skinetrate.com; 4 Department of Pharmaceutics, King Saud University, Riyadh 11454, Saudi Arabia; lalbakr1@ksu.edu.sa; 5 Department of Dentistry, The Sixth Medical Centre of PLA General Hospital, 6 Fucheng Road, Haidian District, Beijing 100048, China; janet_gujing@163.com

**Keywords:** hyaluronic acid, hydrogel, tooth development, epithelial–mesenchymal interaction, human dental pulp stem cells, soft lithography

## Abstract

Tooth loss has been found to adversely affect not just masticatory and speech functions, but also psychological health and quality of life. Currently, teeth replacement options include dentures, bridges, and implants. However, these artificial replacement options remain inferior to biological replacements due to their reduced efficiency, the need for replacements, and the risk of immunological rejection. To this end, there has been a heightened interest in the bioengineering of teeth in recent years. While there have been reports of successfully regenerated teeth, controlling the size and shape of bioengineered teeth remains a challenge. In this study, methacrylated hyaluronic acid (MeHA) was synthesized and microstructured in a hydrogel microwell array using soft lithography. The resulting MeHA hydrogel microwell scaffold resembles the shape of a naturally developing human tooth germ. To facilitate the epithelial–mesenchymal interactions, human adult low calcium high temperature (HaCaT) cells were seeded on the surface of the hydrogels and dental pulp stem cells (DPSCs) were encapsulated inside the hydrogels. It was found that hydrogel scaffolds were able to preserve the viability of both types of cells and they appeared to favor signaling between epithelial and mesenchymal cells, which is necessary in the promotion of cell proliferation. As such, the hydrogel scaffolds offer a promising system for the bioengineering of human tooth germs in vitro.

## 1. Introduction

Teeth are complex organs with distinct architecture, and they serve important masticatory, speech, and aesthetic functions. However, teeth are susceptible to bacterial infection, chemical damage, and mechanical trauma and these are precipitating factors for dental diseases which can ultimately result in loss of dentition [1]. Loss of dentition is a prevalent issue especially in older people and more than 36 million people in the United States are estimated to be edentulous [2]. The functional loss and aesthetic problems resulting from loss of dentition negatively impact psychological health by causing lowered self-confidence [3]. Loss of dentition has also been found to cause a reduction in physical quality of life index [4]. Thus, there is a pressing need for teeth replacement solutions to restore the quality of life of patients following tooth loss.

There are several tooth replacement options currently available—dentures, bridges, and implants [5]. Dentures are removable prosthetic devices that improve masticatory efficiency and aesthetics [6]. Bridges are fixed replacements that are cemented onto and supported by natural teeth. Implants are usually made of titanium and fused into the jawbone to serve as a base for mounting replacement teeth [7]. However, although there has been continuous improvement in these conventional tooth replacement solutions, they remain inferior to natural dentition as these artificial tooth replacement solutions often involve or may result in damage to neighboring teeth and dental tissues [8]. With advances in stem cell biology and tissue engineering, the regeneration of biological teeth has become an attractive method for tooth replacement.

To understand the regeneration of teeth, the natural tooth development process should be understood. One main feature governing the tooth development process is the sequential and reciprocal signaling between the oral epithelium cells and neural crest derived mesenchymal stem cells [9]. The interactions between these two cell types are necessary for the differentiation and subsequent organization of cells into specialized tissues [10].

**Figure 1:**
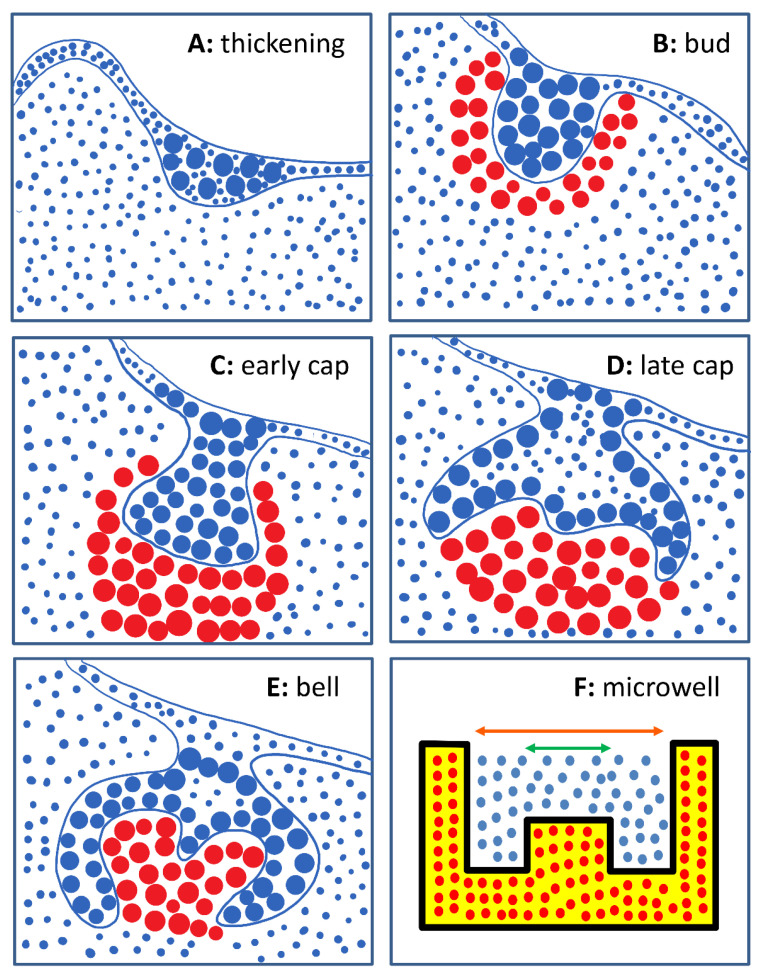
Human tooth development process and schematic representation of a tooth. (**A**) Thickening stage: Proliferation and thickening of oral epithelium; (**B**) Bud stage: Invagination of oral epithelium into mesenchyme and formation of tooth bud; (**C**,**D**) Cap stage: Condensation of mesenchyme around tooth bud, folding and formation of tooth germ; (**E**) Bell stage: Further folding to form tooth crown, differentiation into ameloblasts and odontoblasts. (**F**) Cross-sectional diagram of a single microwell. Yellow area represents methacrylated hyaluronic acid (MeHA) hydrogel structure, blue dots represent human adult low calcium high temperature (HaCaT) cells seeded in the microwell, and red dots represent dental pulp stem cells (DPSCs) encapsulated within the hydrogel. The orange and green arrows represent the outer diameter of the microwell and diameter of the central inlet, respectively.

There are four main stages involved in the natural tooth development process: thickening stage, bud stage, cap stage, and bell stage (Figure 1A–E) [9,11]. In the thickening stage, the oral epithelium proliferates, causing the thickening of the oral epithelium. Development enters the bud stage when the epithelium invaginates into the underlying jaw mesenchyme to form the tooth bud. The surrounding mesenchyme simultaneously condenses around the bud to form the tooth germ. Subsequently, folding begins and the early morphology of a tooth crown can be observed in the cap stage. Finally, in the bell stage, further folding and differentiation of the oral epithelial and mesenchymal cells take place, forming ameloblasts and odontoblasts, respectively. These cells eventually form enamel, dentine, and dental pulp. Root formation and tooth eruption then complete the tooth development process.

Recently, tooth-like structures have been regenerated with the rapid development of tissue engineering and regenerative medicine [12,13]. In 2009, a fully functional murine tooth was successfully regenerated using a tooth-germ engineering method whereby tooth germs were reconstituted using epithelial and mesenchymal cells in vitro before transplantation into an adult mouse jawbone [14]. However, while the bioengineered tooth formed and erupted successfully, it was found to be smaller than natural teeth and the authors were unable to control the crown width and cusp patterning [14]. Although some research has been done on regeneration of animal teeth, there have been no reports of successful regeneration of a complete human tooth.

There are three key elements required in tissue engineering: scaffolds, stem cells, and growth factors [15]. Scaffolds are designed to mimic the extracellular matrix [16]. They not only play an important role in providing biological cues and mechanical stability to the engineered tissues [17], but also help in ensuring ideal placement of cells and proper cell polarization during tissue regeneration [18]. Furthermore, the use of scaffolds has been suggested to reduce tissue regeneration time as they allow the reconstitution of organ germs in the later stages of development [18]. In addition to these important roles, scaffolds should also have controllable biodegradation rates to allow for cell expansion and be sufficiently porous in nature to allow for diffusion of nutrients and metabolites [17]. Hyaluronic acid (HA) hydrogels have been demonstrated to fulfil these requirements [19].

HA is a naturally occurring polymer and a major constituent of the extracellular matrix [20]. HA appears to play an important role in cell proliferation, differentiation, morphogenesis, and migration as environments of highly proliferative cells are often enriched with HA [21]. While HA is quickly degraded by enzymes in vivo, covalent crosslinking of HA polymer chains has been shown to decelerate degradation rates and increase stability [22]. Several studies reported the successful methacrylation of HA to enable photo-crosslinking of HA [23,24]. It has been demonstrated that immediate crosslinking of HA groups upon the addition of the crosslinker before mixing can be completed, resulting in the formation of inconsistent gels [25]. The usage of photopolymerizable methacrylated HA (MeHA) circumvents this problem and presents an advantage over other crosslinking methods.

Two different populations of stem cells are required for tooth engineering, namely, epithelial stem cells and mesenchymal stem cells [26]. The interaction and signaling pathways between these two cell populations initiate, facilitate, and regulate tooth development [26]. Mesenchymal stem cells can be isolated from various sources such as the bone marrow, exfoliated deciduous teeth, and adult dental pulp [27]. Adult dental pulp stem cells (DPSCs) can be easily isolated and have been shown to retain the ability to differentiate into odontoblasts in the presence of the appropriate signals [28]. In addition, DPSCs appear to possess greater potential for dentinogenesis compared to bone marrow derived stem cells and can form dentine-pulp-like complexes in vivo unlike stem cells from exfoliated deciduous teeth [29,30,31,32]. To date, no dental epithelial stem cells (ameloblasts) have been successfully isolated in humans as they undergo apoptosis once tooth eruption occurs [33]. However, there have been reports of the successful induction of human keratinocytes into enamel-secreting ameloblasts in the presence of fibroblast growth factor 8 (FGF-8) [34].

The aim of this study was to develop a microstructured hydrogel tissue culture system to grow tooth germ of a few hundred microns in size. The bioengineered tooth germ can be transplanted for further development. MeHA hydrogel microwell arrays that mimic the natural shape of a developing human tooth germ (Figure 1F) were fabricated by soft lithography. The microwell hydrogel provided a scaffold to compartmentalize the epithelial cells and mesenchymal cells, mimicking the thickening and bud stages of human tooth development. Human adult low calcium high temperature (HaCaT) cells, an immortalized human keratinocyte line, were seeded onto the microwell array and DPSCs were encapsulated within the hydrogels to determine the ability of the scaffold to support the proliferation and growth of dental cells. Finally, a co-culture of HaCaT cells and DPSCs was carried out using the microwell array as a scaffold with the aim of generating human tooth germs.

## 2. Results and Discussion

### 2.1. Nuclear Magnetic Resonance (NMR) Characterization of MeHA

**Figure 2:**
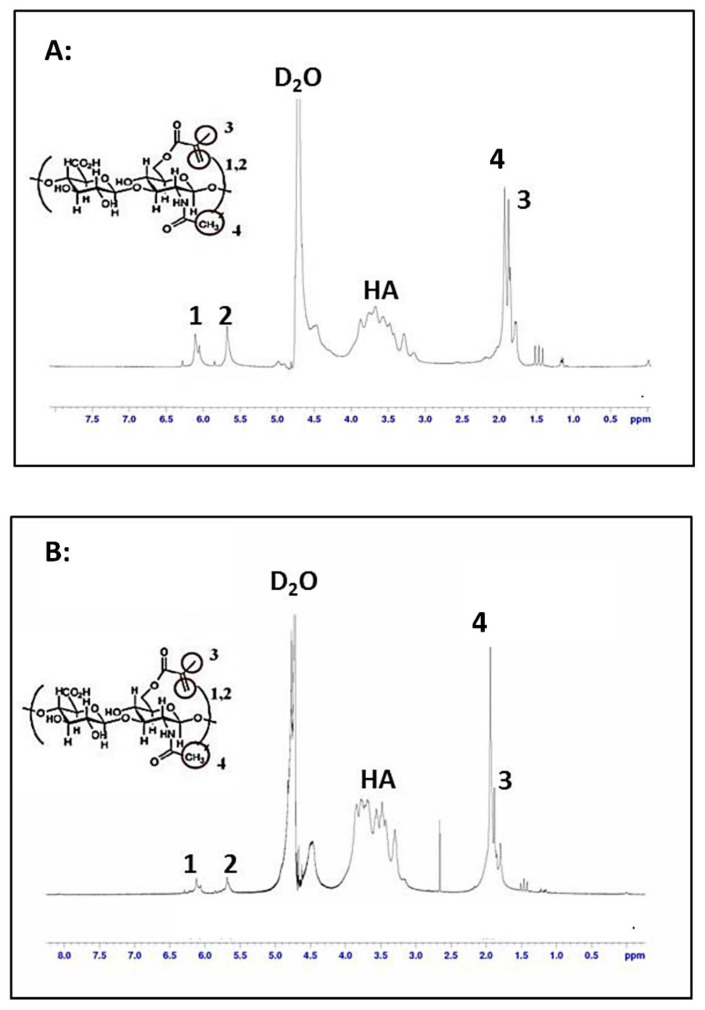
^1^H- Nuclear Magnetic Resonance (NMR) characterization of hyaluronic acid (HA) and MeHA. D_2_O was used as solvent. Peak labelled HA represents hyaluronic acid. Presence of peaks 1, 2, and 3 illustrates the successful methacrylation of HA. Peak 4 represents a methyl group on HA. (**A**) NMR characterization of MeHA synthesized using 20-fold excess of methacrylic anhydride and reaction time of 24 h. Degree of methacrylation (DM) = 77.4%. (**B**) NMR characterization of MeHA synthesized using 6-fold excess of methacrylic anhydride and reaction time of 10 h. DM = 37.8%.

^1^H-NMR spectroscopy was performed on the MeHA synthesized in accordance with the two different reaction conditions to determine the degree of methacrylation (DM) of HA. The degree of methacrylation is defined as the amount of methacryloyl groups per one HA disaccharide repeating unit. ^1^H-NMR spectroscopy of MeHA showed methacrylate peaks at ~6.1, ~5.6, and ~1.85 ppm (Figure 2), confirming the successful methacrylation of HA. The DM was approximated from the ratio of the relative peak integrations of the methacrylate protons (peaks at ~6.1, ~5.6, and ~1.85 ppm) to HA’s methyl protons (peak at ~1.9 ppm) [35]. As controls, the ^1^H-NMR spectra of methacrylic anhydride and hyaluronic acid are shown in supplementary information (SI1).

Manipulation of the reaction time and amount of methacrylic anhydride used for reaction resulted in varying DM of MeHA. It was found that the addition of 20-fold excess of methacrylic anhydride and reaction time of 24 h resulted in a DM of 77.4% (Figure 2A) while a 6-fold excess and reaction time of 10 h resulted in a lower DM of 37.8% (Figure 2B). Increase in DM has been reported to give rise to a greater degree of covalent crosslinking of hyaluronic acid chains, which in turn affects the mechanical and physical properties of the hydrogel synthesized [36]. The hydrogel microwell arrays fabricated using MeHA with higher DM were observed to be stiffer and more brittle in comparison to the hydrogel microwell arrays fabricated using MeHA with lower DM which were softer and more pliable.

The MeHA hydrogel was further characterized with scanning electron microscopy (SI2) and swelling test (SI3). The images exhibited well-defined 3D porous structures with interconnecting channels in the hydrogel. In addition, it was shown that the swelling ratio decreased when the polymer concentration (*w*/*v*) increased from 2.5% to 10%. A concentration of 5% was selected for microwell fabrication, as studies have reported this concentration to be most optimal for cell encapsulation [37]. Although higher concentrations can increase the mechanical stability of hydrogels, high concentrations being more viscous will affect the molding process during fabrication, and may compromise cell viability due to cytotoxicity [38].

### 2.2. Cytotoxicity Test

**Figure 3:**
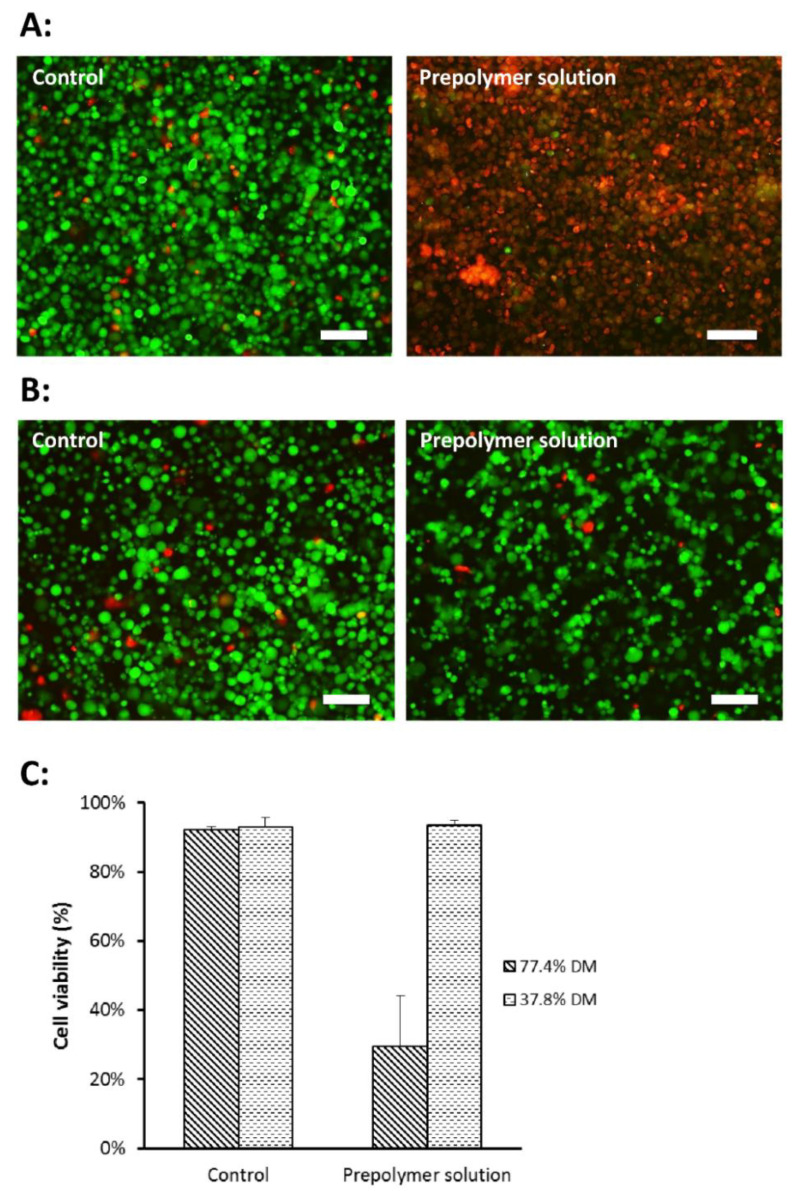
DPSC viability in prepolymer solutions. (**A**) Merged fluorescence images of live/dead assay carried out on cells in control and 77.4% DM MeHA prepolymer solution. Green fluorescence indicates live cells while red fluorescence indicates dead cells. Scale bar represents 100 µm. (**B**) Fluorescence images of live/dead assay carried out on cells in control and 37.8% DM MeHA prepolymer solution. Green fluorescence indicates live cells while red fluorescence indicates dead cells. Scale bar represents 100 µm. (**C**) DPSC viability in control and prepolymer solution of varying DM. Cell viability was calculated by taking fluorescence intensity of live cells over total fluorescence intensity of live and dead cells. Each data point represents the mean ± SD (*n* = 4). Cell viability was 92.4 ± 0.9% and 29.6 ± 1.5% in the control and prepolymer solution, respectively, for 77.4% DM MeHA. Cell viability was 93.0 ± 2.6% and 93.4 ± 1.4% in the control and prepolymer solution, respectively, for 37.8% DM MeHA. (SD = standard deviation).

MeHA of both 77.4% and 37.8% DM were subjected to the cytotoxicity test to determine the toxicity of MeHA on the cells. It was shown that prepolymer solution of 77.4% DM MeHA caused prominent cell toxicity compared with the control (Figure 3A) while prepolymer solution of 37.8% DM MeHA did not (Figure 3B,C). 

The marked cytotoxicity observed with MeHA with higher DM is postulated to be attributed to the significant amount of residual unreacted methacrylic anhydride monomers remaining when a larger excess (20-fold as compared to 6-fold) of methacrylic anhydride was added to achieve the higher DM. Methacrylate monomers have been reported to have a dose dependent cytotoxic effect, causing marked inhibition of cell growth and cell death even at very low concentrations [39]. As a result, MeHA with DM of 37.8% was used to fabricate the hydrogel microwell and for subsequent testing.

If an excess amount of methacrylate anhydride is used in the reaction solution, the unreacted methacrylic anhydride will cause cytotoxicity, if not completely removed from the final product. For future study, the conditions of polymer purification may be optimized according to the extent of extraction of the unreacted components, to minimize cytotoxicity of the polymeric product, for example, by longer time of dialysis or cold precipitation.

### 2.3. Fabrication of Hydrogel Microwell Array

Following photopolymerization of MeHA prepolymer solution by exposure to UV light, solid, soft, and transparent hydrogel microwell arrays were formed. Microwells of two different dimensions were fabricated. The height of the center inlet and depth of the microwell were fixed at 50 μm and 200 μm respectively for both designs. The outer diameters and inlet diameters were varied to produce microwells of different dimensions. The exact outer and inlet diameter for design 1 (⌀ = 200 μm) measured 176.8 ± 3.9 μm and 88.1 ± 4.1 μm. The exact outer and inlet diameter for design 2 (⌀ = 400 μm) measured 397.5 ± 4.3 μm and 164.8 ± 3.3 μm.

### 2.4. DPSC Encapsulation within MeHA Hydrogel Microwell Array

**Figure 4:**
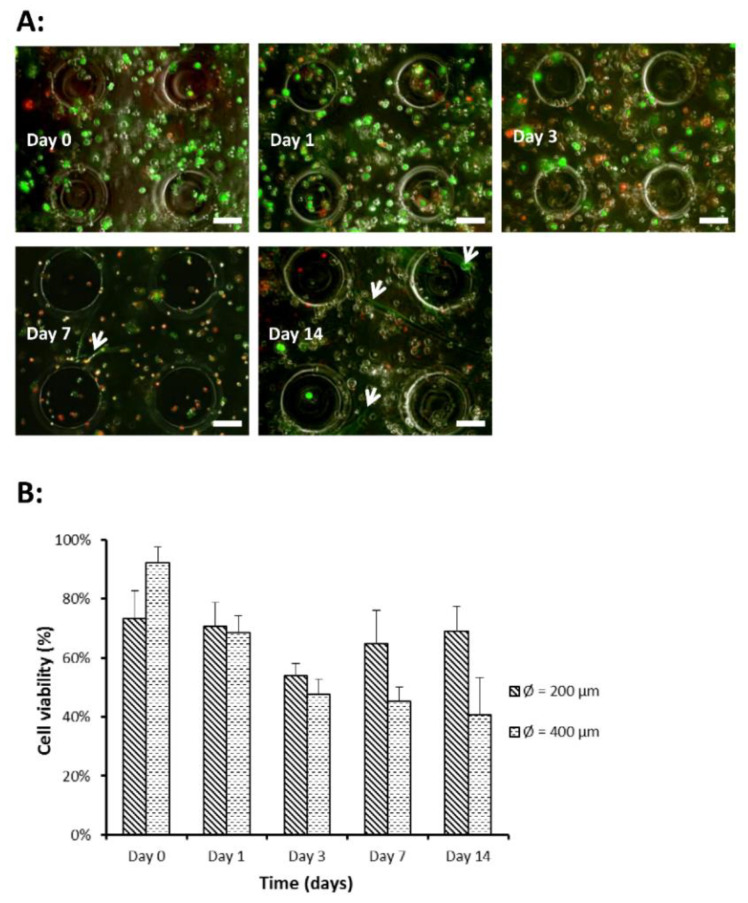
DPSC viability in hydrogel microwell array. (**A**) Merged fluorescence images of live/dead assay performed on encapsulated DPSCs (design 1: ⌀ = 200 μm). Green fluorescence indicates live cells while red fluorescence indicates dead cells. Cell spreading and exhibition of spindle-like morphology is seen on days 7 and 14 (indicated by white arrows). Scale bar = 100 μm. (**B**) Percentage of DPSC viability over time. Cell viability was calculated by taking fluorescence intensity of live cells over total fluorescence intensity of live and dead cells. Each data point represents the mean ± SD (*n* = 6).

Evaluation of cell viability of encapsulated DPSCs in MeHA hydrogel microwell array was conducted to investigate the suitability of MeHA for use as a scaffold material. It was found that the encapsulated DPSCs exhibited cell spreading and adopted a characteristic spindle-like morphology after 7 days in culture (Figure 4A). The live/dead assays demonstrated an initial decreasing trend in cell viability up to day 3 (Figure 4B). The initial decreasing trend in cell viability could be attributed to the presence of free radicals generated from the photoinitiator during the photopolymerization process [40,41]. Free radicals have been reported to react with membrane proteins and DNA and cause cellular damage [42].

### 2.5. HaCaT Cell Seeding in Hydrogel Microwell Array

**Figure 5:**
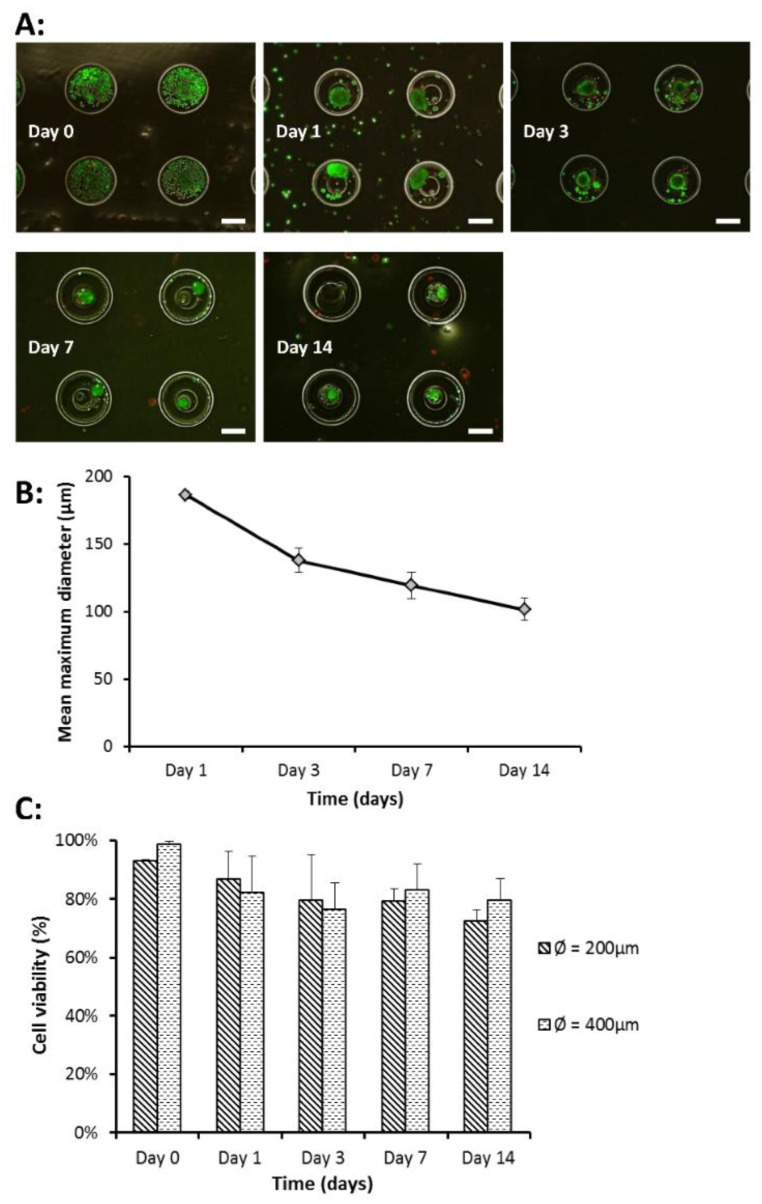
HaCat Cell viability in hydrogel microwell array. (**A**) Merged fluorescence images of live/dead assay performed on seeded HaCaT cells (400 μm microwell). Green fluorescence indicates live cells while red fluorescence indicates dead cells. Day 0 shows successful seeding of HaCaT cells in the microwells. Cell aggregates formed on day 1 and persisted till day 14. Scale bar = 200 μm. (**B**) Mean maximum diameter (μm) of cell aggregates over time (days). Cell aggregates were largest on day 1 at 186 (±2.9) μm on day 1. Aggregate size showed a gradual decreasing trend over the 14 days and final aggregate size was 101 (±8.3) μm on day 14. Each data point represents the mean ± SD (*n* = 4). (**C**) HaCaT cell viability in percentage over time (days). Cell viability was calculated by taking fluorescence intensity of live cells over total fluorescence intensity of live and dead cells. There was no significant difference between cell viability in both microwell designs (*p* = 0.74). Each data point represents the mean ± SD (*n* = 6).

HaCaT cells were seeded in the hydrogel microwell array by the wiping method as described in the method section. The wiping method successfully localized HaCaT cells within the microwells (Figure 5A). Monitoring of cell behavior in microwells at the same location over time revealed that cell aggregates formed on day 1 (Figure 5A) and aggregate size showed a decreasing trend over the 14 days of culture (Figure 5B).

The formation of cell aggregates can be attributed to the low protein absorption and anti-adhesive nature of HA [43], which renders the HA scaffold favorable for aggregate formation. The antifouling property of HA is similar to that of poly (ethylene glycol) diacrylate (PEGDA), which promotes the formation of aggregates but not amenable for cell attachment [44]. Comparison of cell viability over time in microwells of 200 and 400 μm system found that cell viability was comparable in both microwell designs and remained high over 14 days in culture (Figure 5C).

### 2.6. Co-Culture of DPSCs and HaCaT Cells

**Figure 6:**
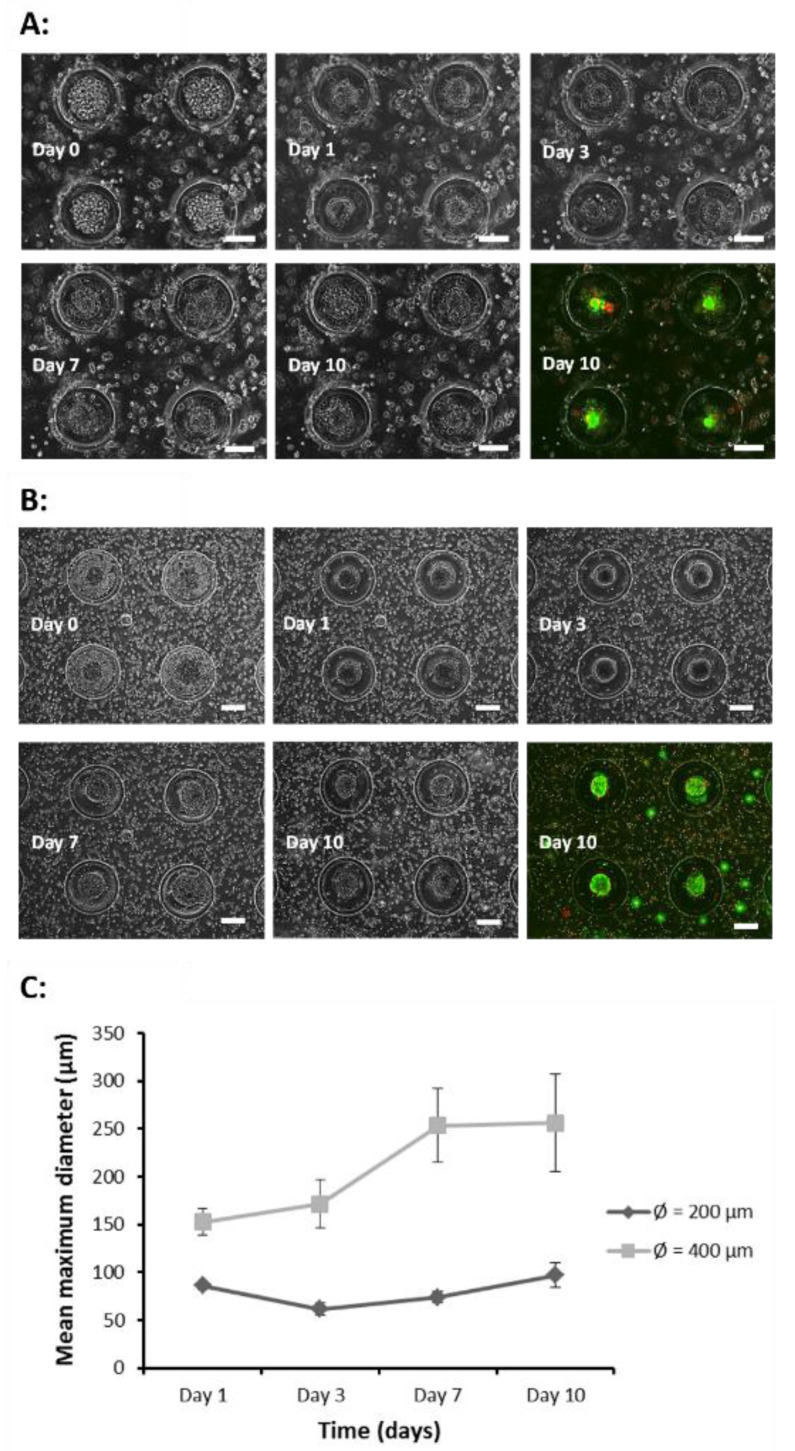
Co-culture of DPSCs and HaCaT cells. Days 0 to 10 represent images of cells in microwells at the same location. Day 0 images indicate successful localization of HaCaT cells within the microwells. Cell aggregates were formed by day 1 and persisted up to day 10. The day 10 image represents the fluorescence images of the live/dead assay. Green fluorescence indicates live cells while red fluorescence indicates dead cells. Scale bars represent 100 μm in (**A**) design 1 and 200 μm in (**B**) design 2. (**C**) Cell aggregate diameter over time.

Co-culture systems using scaffolds of design 1 (⌀ = 200 μm) and 2 (⌀ = 400 μm) were prepared in accordance with the methods as described in the method section. The DPSC laden hydrogel microwell scaffolds were fabricated first, followed by HaCaT cell seeding. In both scaffold designs, HaCaT cells were successfully localized within the microwells and the cells formed aggregates by day 1 and were sustained for up to 10 days (Figure 6A,B). The aggregates in 400 μm microwells were measured to be larger, compared to those in 200 μm microwells, as the number of HaCaT cells trapped in 400 μm microwells was higher than in 200 μm microwells, at the same seeding cell density. The result is consistent with our previous studies [45,46]. The cell aggregate size in the 400 μm microwell reached ~250 μm, which is markedly larger than that in the 200 μm microwell and closer to the size of early-stage tooth germ.

In the co-culture system, the size of the HaCaT cell aggregates stabilized over the 10-day culture (Figure 6C), as compared to the HaCaT cell monoculture (Figure 5C), where it showed a downward trend. The stabilization of aggregate size may be attributed to the presence of signaling interactions between encapsulated DPSCs and HaCaT cells. The signaling molecules such as fibroblasts growth factors have been reported to be critical in promoting the proliferation, differentiation, spreading, and adhesion of cells in tooth development [12,47,48].

In this study, HaCaT cell was used in place of dental epithelial stem cells, due to their unavailability. Recently, the expanding research in human induced pluripotent stem cells shows the promising possibility of regenerating human dental epithelial stem cells (ameloblasts) for use in teeth engineering [49,50]. As such, HaCaT cells may be replaced with induced pluripotent stem cells which offer greater odontogenic potential, for future investigations.

Besides, several improvements can be made to the culture environment to enhance proliferation and differentiation of cells. For example, FGF-8 growth factor has been reported to be critical in mediating epithelial–mesenchymal interactions and inducing ameloblastic differentiation of epithelial cells [34,51]. The incorporation of FGF-8 growth factor in the hydrogel microwell scaffold may be considered in future studies as it may potentially enhance secretion of enamel by HaCaT cells and development of tooth structures. In addition, further studies on the signaling activity of DPSCs and HaCaT cells in the co-culture system can be carried out to form a better understanding of the results generated from this study.

## 3. Conclusions

MeHA was successfully photo-polymerized to fabricate hydrogel microwell arrays resembling the architecture of naturally developing human tooth germs. The hydrogel scaffold was able to support the survival of both DPSCs and HaCaT cells in the co-culture. Consequently, this study illustrates the potential use of MeHA hydrogel microwell arrays as scaffolds to guide cell compartmentalization and development for human tooth bioengineering.

## 4. Materials and Methods

### 4.1. MeHA Synthesis

Methacrylation of HA was performed by adding methacrylic anhydride 94% (Sigma Aldrich) to 1% *w*/*v* of HA (75 kDa, Lifecore, Chaska, MN, USA) in distilled H_2_O (dH_2_O) solution. The amount of methacrylic anhydride added and reaction time were varied to get varying degrees of methacrylation [52]. An excess of methacrylic anhydride, 6-fold and 20-fold relative to primary HA hydroxyl groups, was added and the reactions were carried out for 10 h and 24 h, respectively. The reaction was carried out in the dark at 5 °C. The pH of the 1% *w*/*v* HA solution was adjusted to 8.0 using 5M NaOH (Merck, Darmstadt, Germany) at the beginning of the reaction and then maintained at pH 8 to 9, using 5M NaOH for the entire duration of the reaction. Subsequently, the solution was dialyzed by using a cellulose dialysis tubing with the molecular weight cut-off of 11035 (Sigma Aldrich, Singapore) for 48 h in dH_2_O and freeze-dried using a lyophilizer (Labconco, Kansas City, MO, USA) for 72 h. The lyophilized MeHA was stored at −20 °C before use.

### 4.2. NMR Characterization of MeHA

The DM of HA was determined with ^1^H-NMR spectrometry. An MeHA in deuterium oxide (Sigma Aldrich, Singapore) solution 3% *w*/*v* was used for the NMR analysis. The spectra of MeHA were obtained by using a 400-MHz NMR spectrometer. To find the DM, a ratio of the relative peak integrations of the methacrylate protons (peaks at 6.1 ppm, 5.6 ppm, and 1.85 ppm) and HA’s methyl protons (peak at ~1.9 ppm) was calculated [53].

### 4.3. MeHA Prepolymer Solution Preparation

The 0.05% *w*/*v* 2-hydroxy-4-(2-hydroxy-ethoxy)-2-methyl-propiophenone (HHEMP) photoinitiator solution (33 wt% Irgacure 2959; Ciba, Timonium, MD, USA) was prepared by dissolving HHEMP in phosphate buffered saline (PBS) diluted to 1× concentration (Vivantis, Selangor, Malaysia) at 70 °C. The HHEMP photoinitiator solution was sterilized by filtration with a 0.2 µm membrane syringe filter (Pall Corporation, Port Washington, NY, USA). As indicated by the company, a pore size of 0.2 µm was suitable for producing sterile filtrate. In addition, the prepolymer solution is further sterilized when being subjected to UV irradiation. MeHA prepolymer solution was then prepared by dissolving lyophilized MeHA at a concentration of 5% *w*/*v* in 0.05% *w*/*v* HHEMP photoinitiator solution.

### 4.4. Fabrication of Polydimethylsiloxane (PDMS) Lithographical Stamp

**Figure 7:**
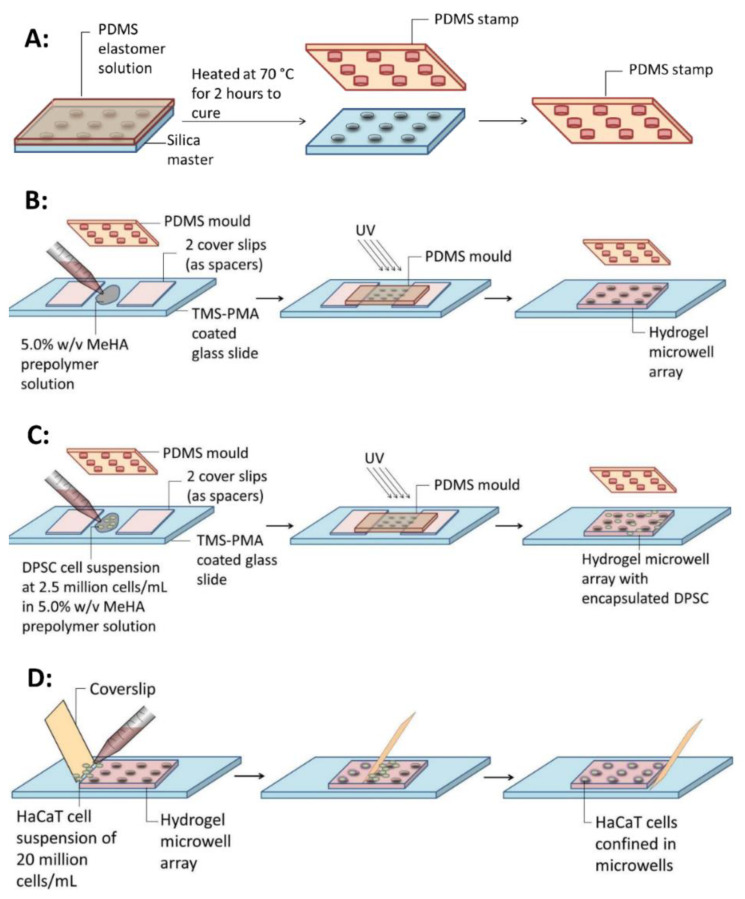
The schematic representation of microfabrication and cell seeding. (**A**) Fabrication of polydimethylsiloxane (PDMS) stamp using a SU-8 silicon master. (**B**) Fabrication of MeHA hydrogel microwell array by casting the photo-polymerization of 5.0% *w*/*v* MeHA prepolymer solution onto the PDMS stamp. (**C**) Encapsulation of DPSCs in MeHA hydrogel microwell array by suspending the cells inside prepolymer solution. The greyish spots represent the DPSCs encapsulated inside the hydrogels. (**D**) Seeding of HaCaT cells in microwells using a wiping method.

The PDMS lithographical stamp was fabricated by mixing the silicone elastomer base solution and curing agent of Sylgard 184 (Dow Corning Corporation, Midland, MI, USA) at the ratio of 10:1. The viscous solution was degassed to remove the bubbles in a vacuum chamber (Thermo Fisher, Waltham, MA, USA). Afterwards, the solution was poured onto a patterned SU-8 silicon master and then kept at 70 °C for ~2 h for curing. The PDMS stamps were peeled from the silicon master after being cured (Figure 7A). Two types of microwells were fabricated. For Design 1, the diameters of microwell and the center inlet were 66 μm and 200 μm. For Design 2, the diameters of microwell and the center inlet were 133 μm and 400 μm.

### 4.5. Hydrogel Microwell Array Fabrication

To bond the hydrogels onto the glass surface, the following procedure was carried out. The microscopic glass slide was soaked in 0.4% *v*/*v* 3-(trimethoxysilyl) propyl methacrylate (TMS-PMA) (Sigma Aldrich, Singapore) for 12 h to provide bonding sites on the glass surface [54]. The glass side was then rinsed with water and dried at 70 °C for 2 h. Two coverslips were stacked up as spacers on the glass slide. Then the PDMS stamp was placed on top of the two coverslips (Figure 7B). To make the PDMS surface wettable, the PDMS stamp was treated using an oxygen plasma cleaner (Harrick Plasma, Ithaca, NY, USA) for 3 min. Afterwards, 50 μL of 5.0% *w*/*v* MeHA prepolymer solution was carefully added into the gap between the PDMS stamp and glass slide using a micropipette. The prepolymer solution was then exposed to ultraviolet (UV) radiation of 4.3 W/cm^2^ for 40 s at 4 cm to the light source OmniCure s2000 (Excelitas Technologies, Waltham, MA, USA). After exposure, the PDMS stamp was peeled off from the surface, coverslip spacers were removed, and the formed hydrogel microwell array was placed in 10 mL of PBS solution.

The fabrication of hydrogel microwell arrays containing DPSCs is presented separately in Section 2.6.

### 4.6. Tissue Culture

For tissue culture, the cells were manipulated under aseptic conditions in a biosafety cabinet and maintained in a humidified incubator filled with 5% carbon dioxide at 37 °C. The culture media for HaCaT cells were Dulbecco’s modified Eagle medium (Thermo Fisher, Waltham, MA, USA), containing 10% *v*/*v* fetal bovine serum (Thermo Fisher, Waltham, MA, USA), penicillin-Streptomycin solution with 10,000 units penicillin and 10 mg streptomycin / mL (Thermo Fisher, Waltham, MA, USA). The culture media for DPSC (AllCells, Alameda, CA, USA) were α-Minimum essential medium (Thermo Fisher, Waltham, MA, USA), containing 10% *v*/*v* mesenchymal stem cell qualified fetal bovine serum (Life Technologies, Singapore), penicillin-streptomycin solution with 10,000 units penicillin and 10 mg streptomycin/mL (Thermo Fisher, Waltham, MA, USA). All components of culture media were filtered using 0.22 μm pore Corning filter units (Dow Corning Corporation, Midland, MI, USA). All cells were cultured in T75 cell culture flasks (Dow Corning Corporation, Midland, MI, USA) and the culture media was changed every 3 days.

### 4.7. Cytotoxicity Test

The MeHA powder was added in PBS to form a 5% *w*/*v* solution. DPSCs were then suspended in the solution at 2.5 million cells/mL. A control was prepared by adding DPSCs in PBS at 2.5 million cells/mL. Cell viability assays were carried out on cells in the control and MeHA pre-polymer solution. Cell suspension (50 μL) was used for each viability assay, using the LIVE/DEAD^®^ Viability/Cytotoxicity Assay Kit (Thermo Fisher, Waltham, MA, USA).

### 4.8. DPSC Encapsulation inside the Hydrogel

Fabrication of DPSC-laden hydrogel microwell arrays was carried out by suspending DPSCs in 5% *w*/*v* MeHA prepolymer solution to form a 2.5 million cells/mL cell suspension. The resulting prepolymer solution was then subjected to the conditions specified in Section 2.5 for the fabrication of the hydrogel microwell array (Figure 7C). The hydrogels with encapsulated DPSCs were maintained in MEM α culture media containing 10 % mesenchymal stem cell qualified fetal bovine serum (Life Technologies, Singapore) in a 95% air/5% CO_2_ humidified incubator at 37 °C and the culture media was changed every 3 days. Cell viability assays were performed in triplicate on days 0, 1, 3, 7, and 14 after encapsulation.

### 4.9. Cell Seeding

Seeding of HaCaT cells in the fabricated hydrogel microwells were carried out using a previously developed wiping method [55] (Figure 7D). The fabricated hydrogel microwell arrays were gently patted dry with UV-sterilized Kimwipes (Kimberly-Clark, Irving, TX, USA). Then 20 μL of HaCaT cell suspension (20 million cells/mL) was added to the edge of a coverslip and wiped across the microwells using the coverslip. Once spread onto the glass slide, the cells in the suspensions settled into the microwells in a few minutes because of gravitational forces. The microwell arrays seeded with HaCaT cells were maintained in culture media in a 95% air/5% CO_2_ humidified incubator at 37 °C and the media was changed every 3 days. Cell viability assays were performed in triplicates on days 0, 1, 3, 7, and 14 after seeding, using a Nikon Eclipse T*i* fluorescence microscope (Nikon, Tokyo, Japan).

### 4.10. Co-Culture of DPSCs and HaCaT Cells

DPSC-laden hydrogel microwell arrays were prepared following the method specified in Section 2.6. The DPSC laden hydrogel microstructures were placed in cell culture dishes (Greiner Bio-one, Frickenhausen, Germany) containing 12 mL of PBS solution immediately after fabrication to remove free radicals generated during the photo-polymerization process. HaCaT cells were then seeded in the DPSC-laden hydrogel microwell arrays following the method specified in Section 4.9. The hydrogels with encapsulated DPSCs were maintained in the culture media in a 95% air/5% CO_2_ humidified incubator at 37 °C and the media was changed every 3 days. Same location observations were conducted on days 0, 1, 3, 7, and 10, while cell viability assays were conducted in triplicate on day 10.

### 4.11. Cell Viability Assay

The LIVE/DEAD^®^ Viability/Cytotoxicity Assay Kit (Thermo Fisher, Waltham, MA, USA) was used to prepare the testing solution, according to the manufacturer’s notes. The culture medium was removed before addition of 50 uL of assay solution on the hydrogel microwell arrays. The hydrogel microwell arrays were then incubated with the solution for 30 min at 37 °C. Live cells fluoresced green at a wavelength of 495 nm excitation, due to metabolic conversion of non-fluorescent calcein-AM to fluorescent calcein by intracellular esterase. At 590 nm excitation, dead cells with compromised cellular membranes fluoresced red, as ethidium homodimer crossed the cell membrane to bind DNA. The fluorescent images were taken with a Nikon Eclipse T*i* microscope (Nikon, Tokyo, Japan) and cell fluorescence intensities were analyzed with ImageJ software. The cell viability was computed as the ratio of fluorescence intensity of live cells to total intensity of live and dead cells.

### 4.12. Statistical Analysis

The Student’s t-test and one-way analysis of variance (ANOVA) were used to determine the statistical significance of the differences between results. A *p*-value of less than 5% was considered significantly different.

## Supplementary Materials

The following are available online at https://www.mdpi.com/article/10.3390/gels7030123/s1. SI1. The proton NMR spectrum of methacrylic anhydride and hyaluronic acid. SI2. The scanning electron microscope (SEM) images of MeHA hydrogel. SI3. Mass swelling ratio of MeHA hydrogel.
