# Supplementary material for: Microstructured Hyaluronic Acid Hydrogel for Tooth Germ Bioengineering
Source: Gels. 2021 Aug 18;7(3):123. doi: 10.3390/gels7030123 (PMC8395742; doi:10.3390/gels7030123)
Supplement: Supplementary file 1 [file gels-07-00123-s001.zip › gels-1310571-supplementary.pdf]

gels

MDPI

Microstructured Hyaluronic Acid Hydrogel for Tooth Germ Bioengineering

Supplementary Information (SI)

SI1. The proton NMR spectrum of methacrylic anhydride and hyaluronic acid.

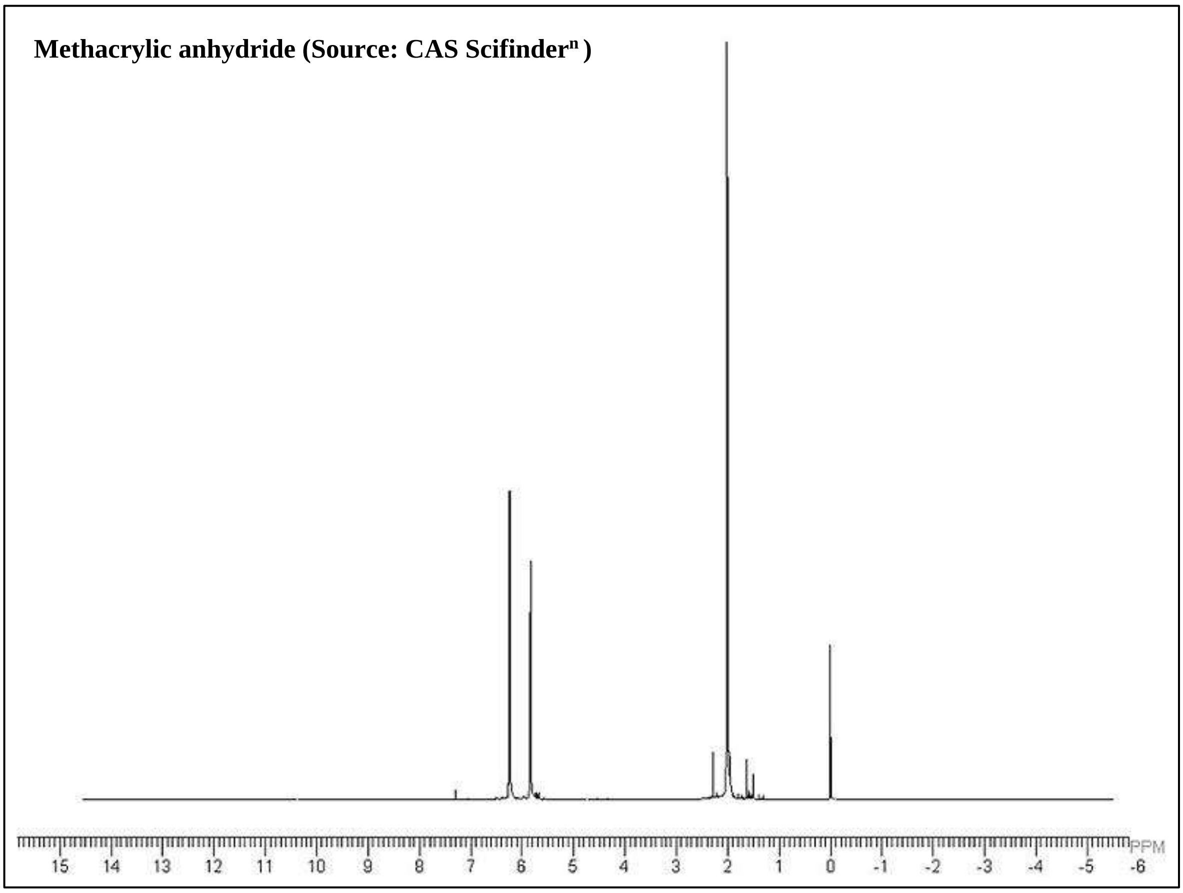

Hyaluronic acid (experimental data)

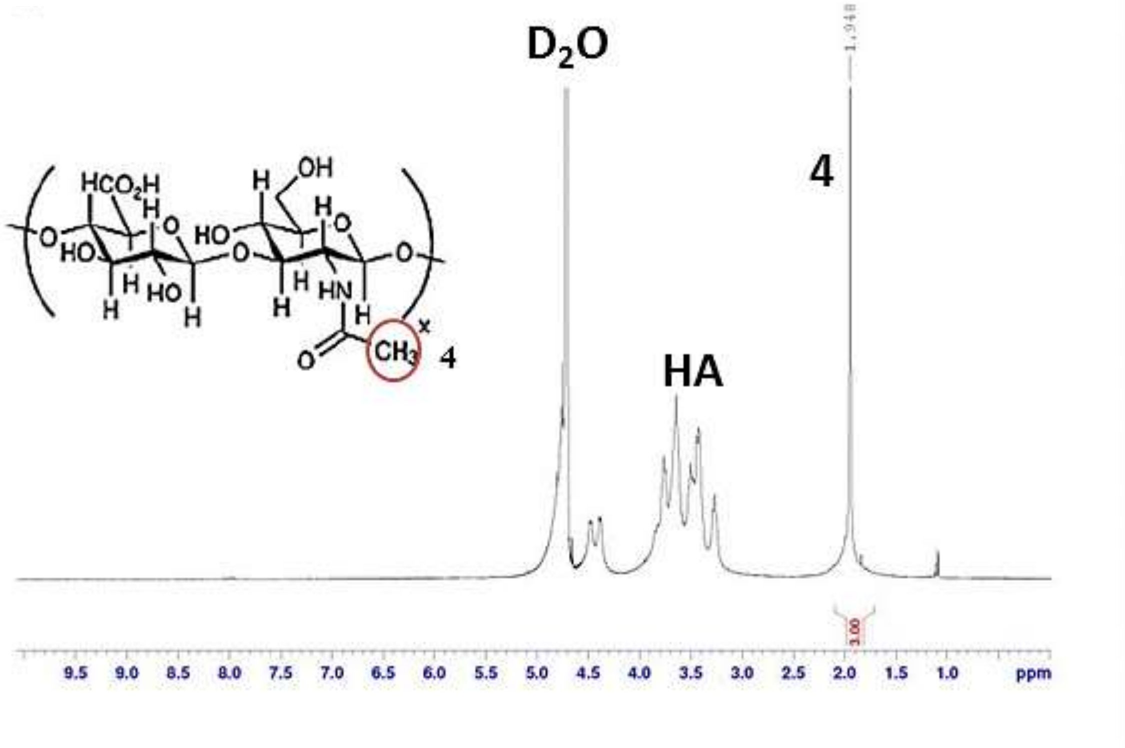

SI2. The scanning electron microscope (SEM) images of methacrylated hyaluronic acid (MeHA) hydrogel at various concentrations (250 × magnification). Scale bar = 100 μm.

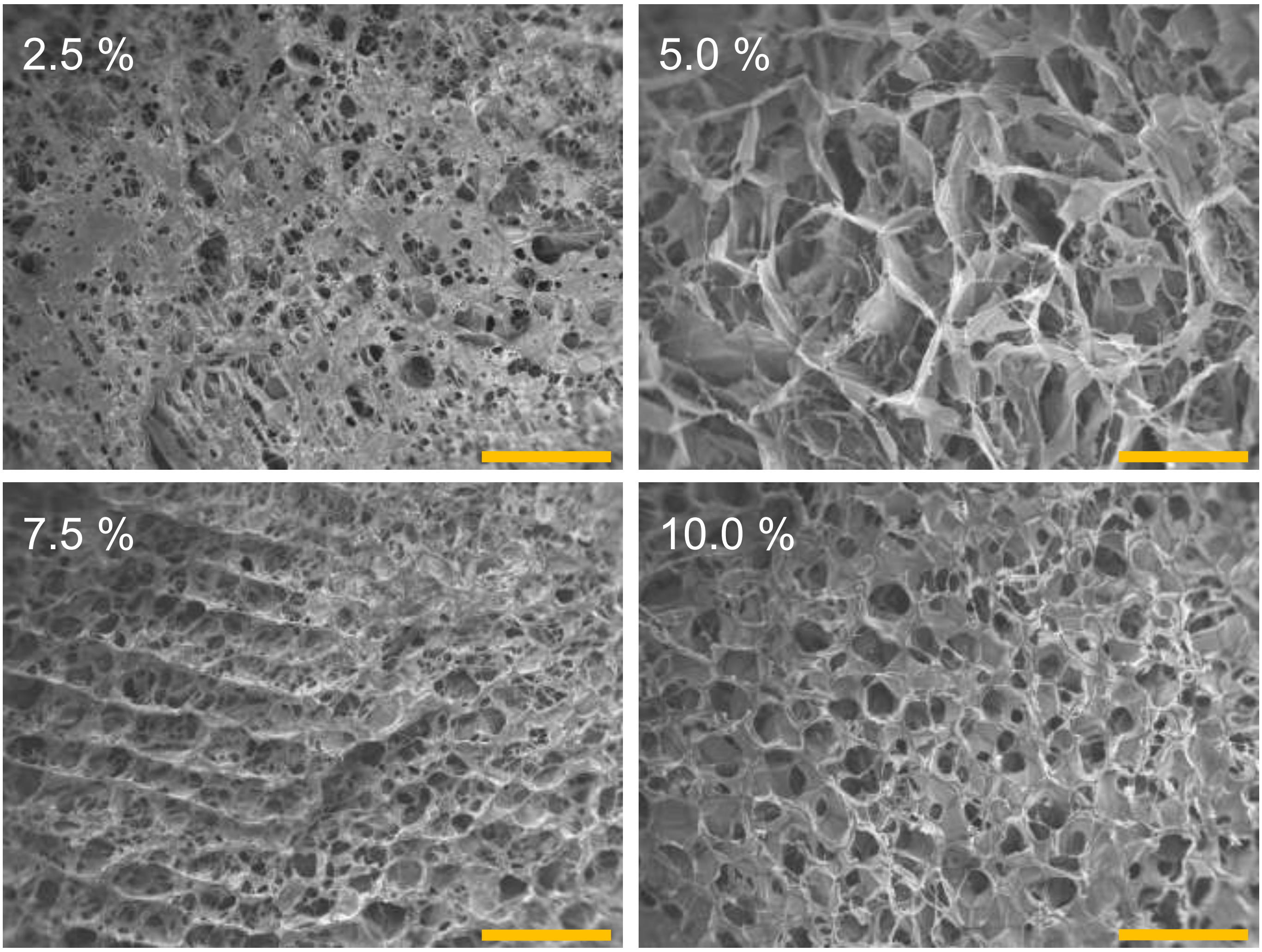

The prepolymer solution containing MeHA of 2.5, 5, 7.5 and 10%, respectively, was UV-crosslinked and then lyophilized, prior to SEM imaging. The degree of methacrylation (DM) of the MeHA was 37%. (The details of prepolymer solution preparation can be found in the main text: section 4.3. MeHA prepolymer solution preparation). All hydrogels showed well-defined 3D porous structures with interconnecting channels.

SI3. Mass swelling ratio of MeHA hydrogel at various DM and concentrations.

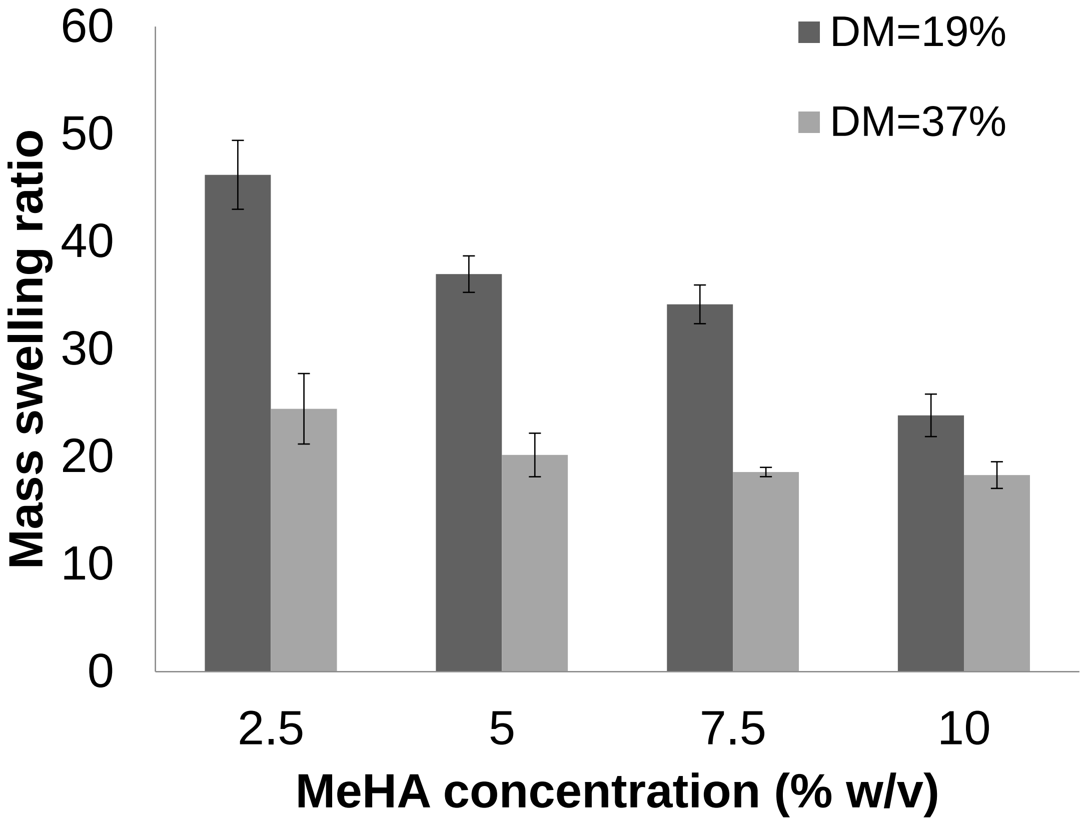

To measure the mass swelling ratio, 4 different concentrations (*w*/*v*) of the MeHA prepolymer solution were prepared, ranging from 2.5 %, 5.0 %, 7.5 %, to 10.0 %, containing the photoinitiator in each solution. Afterwards, two cover slips were used as the spacer to support a third cover slip, which was placed on top of the 2 cover slips, on a glass slide. Using a micropipette, 100 µl of prepolymer solution was added to the gap, which was subjected to UV irradiation to cure the MeHA prepolymer solution. Then the cured hydrogel was removed from the glass slide and incubated in PBS solution. After a period of 24 hours at 37° C, the hydrogel was removed from PBS and the mass was weighed. The hydrogel was then lyophilized and weighed again to find the mass of polymer. To calculate the swelling ratio, the mass of hydrogel was divided by the mass of dry polymer.

It was shown that the swelling ratio decreased when the polymer concentration increased. Jason and colleagues also showed the reduction in the volumetric swelling ratio caused by increase in the concentration of MeHA [1]. At a given polymer concentration, the swelling ratio was higher at lower degree of methacrylation. The result was in consistence with previous studies, showing that fewer crosslinking sites resulted in a lower crosslink density, which in turn caused higher swelling ratio [2].

References

1. Burdick, J.A.; Chung, C.; Jia, X.; Randolph, M.A.; Langer, R. Controlled degradation and mechanical behavior of photopolymerized hyaluronic acid networks. *Biomacromolecules* **2005**, *6*, 386-391, doi:10.1021/bm049508a.
2. Leach, J.B.; Bivens, K.A.; Collins, C.N.; Schmidt, C.E. Development of photocrosslinkable hyaluronic acid-polyethylene glycol-peptide composite hydrogels for soft tissue engineering. *J Biomed Mater Res A* **2004**, *70*, 74-82, doi:10.1002/jbm.a.30063.
